# A specific mode of microsatellite instability is a crucial biomarker in adult T-cell leukaemia/lymphoma patients

**DOI:** 10.1007/s00432-016-2294-1

**Published:** 2016-10-25

**Authors:** Kaname Miyashita, Kei Fujii, Kenichi Taguchi, Mototsugu Shimokawa, Mitsuaki A. Yoshida, Yasunobu Abe, Jun Okamura, Shinya Oda, Naokuni Uike

**Affiliations:** 1 Clinical Research Institute, National Kyushu Cancer Center, Fukuoka, 811-1395 Japan; 2 Department of Hematology, National Kyushu Cancer Center, Fukuoka, 811-1395 Japan; 3 Department of Medicine and Bioregulatory Science, Graduate School of Medical Sciences, Kyushu University, Fukuoka, 812-8582 Japan; 4 Department of Surgery and Oncology, Graduate School of Medical Sciences, Kyushu University, Fukuoka, 812-8582 Japan; 5 Department of Radiation Biology, Institute of Radiation Emergency Medicine, Hirosaki University, Aomori, 036-8560 Japan

**Keywords:** Microsatellite instability, DNA mismatch repair, Adult T-cell leukaemia/lymphoma, Chemotherapy, Drug resistance, Biomarker

## Abstract

**Purpose:**

Microsatellite instability (MSI) has been a long-standing biomarker candidate for drug resistance in tumour cells. Despite numerous clinical studies, the data in the literature are not conclusive. The complexity of the MSI phenomenon in some malignancies may, at least partly, account for the discrepancy. In addition, methodological problems are also pointed out in the assay techniques. We previously established a unique fluorescent technique in which the major methodological problems in conventional assays are overcome. Application of this technique has revealed two distinct modes of microsatellite alterations, i.e. Type A and Type B. More importantly, we demonstrated that Type A MSI is the direct consequence of defective DNA mismatch repair (MMR) that causes cellular resistance against antineoplastic agents.

**Method:**

We first applied this technique to adult T-cell leukaemia/lymphoma (ATLL).

**Results:**

The MSI phenomenon was indeed observed in ATLLs (4/20, 20%). Intriguingly, the observed microsatellite alterations were invariably Type A, which implies that the tumours were MMR-defective. Indeed, clinical outcomes of patients with these MSI^+^ tumours were significantly worse. Furthermore, multivariate analysis revealed that Type A MSI is an independent prognostic factor.

**Conclusion:**

These observations strongly suggest the possibility of Type A MSI as a prognostic and potentially predictive biomarker in ATLL.

## Introduction

Adult T-cell leukaemia/lymphoma (ATLL) is a mature T-lymphocyte neoplasm with a causative link to retroviral infection by human T-cell leukaemia virus 1 (HTLV-1) (Matsuoka and Jeang 2007; Uchiyama 1997; Uchiyama et al. 1977; Verdonck et al. 2007), which is endemic in geographically limited areas including south-western Japan, sub-Saharan Africa, the Caribbean basin and South America (Matsuoka and Jeang 2007; Verdonck et al. 2007). HTLV-1-infected populations are estimated to exceed ten million around the world. The majority of the infected individuals however remain asymptomatic throughout their lives, and a small subpopulation, less than 5%, develops ATLL (Arisawa et al. 2000; Matsuoka and Jeang 2007). Among the four clinical subtypes (Shimoyama 1991), acute and lymphoma types exhibit an extremely poor patient prognosis, primarily because of resistance to antineoplastic agents. The median survival time is approximately one year in patients treated with conventional chemotherapy (Tsukasaki et al. 2007; Yamada et al. 2001). Although target-based therapies using an anti-CCR4 antibody are now regarded as promising (Ishida et al. 2012; Yamamoto et al. 2010), allogeneic haematopoietic stem cell transplantation (HSCT) has been tested during this decade (Hishizawa et al. 2010; Jabbour et al. 2011; Okamura et al. 2005; Utsunomiya et al. 2001), and remission has been reported in some cases. These favourable results of HSCT are now discussed in connection with graft-versus-host reactions (Kanda et al. 2012; Yonekura et al. 2008). In addition to factors to modulate the host immune response, those leading to an extreme drug resistance in tumours also need to be discussed. Biomarkers to predict response to chemotherapy may be prerequisite for personalised approaches to more effective treatment of ATLL patients.

Microsatellite instability (MSI), i.e. somatic destabilisation of microsatellite DNA sequences comprising short reiterated motifs, is regarded as reflecting defective DNA mismatch repair (MMR) in tumour cells. MMR is an important repair system coupled with DNA replication that counteracts replication errors caused by DNA polymerases (Jiricny 2006; Modrich 2006). It is now widely known that MMR deficiency leads to cellular resistance against various antineoplastic agents (Jiricny 2006; Karran 2001), presumably due to avoidance of catastrophic repair reactions on the genome. MSI has therefore been regarded as a promising candidate for a predictive biomarker in the field of oncology. Numerous studies have been done to elucidate its role in tumour response after chemotherapy, particularly in colorectal cancer patients treated with 5-fluorouracil (5-FU). However, the clinical research data reported in the literature lack unity (Guastadisegni et al. 2010), whereas 5-FU resistance in MMR-defective cells has been established in vitro (Carethers et al. 1999; Tokunaga et al. 2000a). This may be, at least partly, due to the complexity of the MSI phenomenon in colorectal cancer. In this malignancy, microsatellite alterations appear to be comprised of several subcategories, depending on the involved microsatellite sequences, the repair defects and the tumourigenesis pathways. The classification of MSI has in fact been controversial. In 1997, the National Cancer Institute (NCI)-sponsored workshop, ‘Microsatellite Instability and RER Phenotypes in Cancer Detection and Familial Predisposition’ (Boland et al. 1998), recommended that the MSI^+^ phenotype should be classified into two different grades, i.e. MSI-H (high) (≥20%) and MSI-L (low) (<20%), according to the frequencies of changes in a defined set of microsatellite markers. This MSI grading established by NCI has been widely used since then and, according to the MSI grades, the two mutually exclusive pathways have been hypothesised in colorectal tumourigenesis. However, several reports suggest that this distinction may be an oversimplification (Goel et al. 2003; Hawkins et al. 2001; Jass et al. 2002; Young et al. 2001). In addition, the techniques used for microsatellite analyses have been in confusion, and several methodological problems are indeed pointed out in the conventional assay techniques (Maehara et al. 2001). We previously established a unique fluorescent technique in which the major methodological problems are overcome (Oda et al. 1997). Application of this technique has revealed a number of previously unrecognised aspects of MSI in human cancers. In particular, we observed two distinct modes of microsatellite alterations, i.e. Type A and Type B (Oda et al. 2005). More importantly, we found that Type A MSI is the direct consequence of defective MMR, and that Type B instability may involve molecular abnormalities in addition to the repair defect. The existence of these different modes of MSI has shed light on the complexities in the relationship between MMR defects and MSI in human cancers, and also in the variety of genomic instability underlying tumourigenesis. Thus, the microsatellite stability-based designation of the MMR status appears more difficult than hitherto expected.

We have applied our fluorescent technique to various human malignancies including colorectal (Ikeda et al. 2001), endometrial (Eto et al. 2016), gastric (Sakurai et al. 2007), oesophageal (Araki et al. 2004) and breast (Tokunaga et al. 2000b) cancers. We have also analysed the microsatellite stability in several haematological malignancies using this technique, and found the MSI phenomenon in non-Hodgkin lymphoma patients (Miyashita et al. 2008). Intriguingly, alterations observed in these microsatellite-unstable lymphomas were uniformly Type A, which implies that the tumours were MMR-defective (Oda et al. 2005). Indeed, response to chemotherapy was significantly worse in patients with MSI^+^ tumours, and clinical outcomes were also less favourable in this population. These findings strongly suggest the possibility of MSI as a prognostic and, simultaneously, predictive biomarker in haematological malignancies. Although the predominance of Type A MSI in the lymphoma genome is of potential interest in connection with lymphomagenesis, the role of MSI merits particular attention in the clinical context. In the present study, we have applied our fluorescent technique to another haematological malignancy, ATLL, in which chemotherapy has faced limits and difficulties, and, therefore, predictive biomarkers are expected to improve the treatment strategies. Here, we report that Type A MSI is indeed observed in tumours arising in ATLL patients, and that this tumour phenotype was tightly connected to poor clinical outcomes of the patients. Our findings strongly suggest that Type A MSI is a prognostic and potentially predictive biomarker in ATLL patients.

## Materials and methods

### Patients and tissue/cell specimens

Twenty consecutive patients diagnosed as ATLLs and treated in the Department of Hematology, National Kyushu Cancer Center from 1997 to 2003 were enrolled in this study. Patients whose tumour and corresponding normal tissue specimens were not available were excluded. Patient characteristics are summarised in Table 1. Men and women were fourteen and six, respectively. Age at diagnosis ranged from 36 to 81 years old, with a mean of 64 years old. The disease subtype of each tumour was determined according to Shimoyama criteria of ATLL classification (Shimoyama 1991). All the patients had no history of other malignancies that is consistent with genetic predisposition such as Lynch syndrome. The majority of the patients received modified EPOCH (mEPOCH) initial chemotherapy, in which cyclophosphamide is replaced with carboplatin in the classical EPOCH regimen (Ishitsuka and Tamura 2014; Wilson et al. 1993), and some in combination with intrathecal injection or radiotherapy (Table 1). In some elderly patients, the CHOP (Fisher et al. 1993) or the oral sobuzoxane plus etoposide (PVP) (Kagami et al. 1996) therapies were also used. Response to initial treatments was judged according to the International Consensus Meeting criteria (Tsukasaki et al. 2009). Tumour tissue specimens were sampled from lymph nodes, skin or other soft tissues. Tumour cells were also collected from peripheral blood or bone marrow which contains tumour cells more than 30% of total mono-nuclear cell number. Corresponding normal tissue specimens were also collected from bone marrow or peripheral blood without tumour cells (i.e. less than 10% of mono-nuclear cells). Ethical approval was obtained from the IRB of National Kyushu Cancer Center. Written informed consent for studies was obtained from each patient.

**Table 1:** Microsatellite alterations observed in 20 ATLL patients

Patient code	Clinicopathological variable												Microsatellite				
	Subtype	Stage	Age	Gender	PS	High LDH	High Ca	>3 lesions	Initial treatment	Response	HSCT	Outcome	D2S123	D5S107	D10S197	D11S904	D13S175
ATL01	L	IV	60	M	1	+	−	+	mEPOCH	CR	−	Dead	−	MSI	−	−	−
ATL02	L	II	68	M	0	−	+	−	mEPOCH	CR	−	Alive	LOH	−	LOH’	−	−
ATL03	A	IV	36	F	1	+	−	+	mEPOCH + RT + IT	PR	+	Alive	−	−	−	−	−
ATL04	L	III	67	M	1	+	−	−	mEPOCH + RT	PR	−	Dead	−	−	LOH	−	−
ATL05	A	IV	63	M	1	+	−	+	mEPOCH	CRu	−	Alive	LOH	−	LOH’	−	−
ATL06	L	III	59	F	0	+	−	−	mEPOCH	PR	−	Alive	LOH	−	−	−	−
ATL07	L	III	74	M	4	+	+	+	mEPOCH	PD	−	Dead	MSI	−	−	−	−
ATL08	A	IV	81	F	2	+	−	+	PVP	PR	−	Dead	−	−	−	−	LOH’
ATL09	A	IV	56	M	0	+	+	+	mEPOCH	PD	−	Dead	MSI	−	−	−	−
ATL10	A	IV	48	F	1	+	+	+	mEPOCH + IT	CR	+	Alive	LOH	−	LOH	−	−
ATL11	A	IV	65	F	2	+	−	+	mEPOCH	PD	+	Alive	−	−	−	−	−
ATL12	L	IV	75	M	2	+	−	+	mEPOCH	PR	−	Dead	−	−	−	−	LOH’
ATL13	L	II	77	M	0	+	−	−	CHOP	CR	−	Alive	−	−	−	−	−
ATL14	L	IV	58	M	2	+	−	+	mEPOCH	PR	+	Dead	−	−	−	−	−
ATL15	L	III	62	M	2	+	−	+	mEPOCH	PD	−	Dead	−	−	−	−	−
ATL16	A	IV	70	M	4	+	+	+	mEPOCH	NE	−	Dead	−	MSI	−	−	MSI
ATL17	L	II	73	M	3	+	−	−	mEPOCH	PR	−	Dead	−	−	−	−	−
ATL18	L	III	55	M	1	+	+	−	mEPOCH	PR	+	Alive	−	−	−	−	−
ATL19	A	IV	57	F	4	+	+	+	mEPOCH	PR	+	Dead	−	−	−	−	−
ATL20	L	IV	71	M	3	+	+	+	mEPOCH	PR	−	Dead	LOH	−	−	LOH’	−

*A* acute type, *CHOP* cyclophosphamide, doxorubicin, vincristine and prednisolone, *CR* complete remission, *CRu* uncertified complete remission, *F* female, *High Ca* hypercalcaemia, *High LDH* elevation of serum lactate dehydrogenase, *HSCT* allogeneic haematopoietic stem cell transplantation, *IT* intrathecal injection, *L* lymphoma type, *LOH* loss of heterozygosity, *LOH′* alterations theoretically indistinguishable between Type A MSI and LOH, *M* male, *mEPOCH* etoposide, prednisolone, vincristine, doxorubicin and carboplatin, *MSI* microsatellite instability, *NE* not evaluated, *PD* progressive disease, *PR* partial remission, *PS* Eastern Cooperative Oncology Group performance status, *PVP* sobuzoxane and etoposide, *RT* radiotherapy, *Stage* Ann Arbor stage

### DNA extraction

Tumour specimens were lysed in digestion buffer (10 mM Tris–Cl pH 8.0, 0.1 M EDTA pH 8.0, 0.5% SDS, 20 µg/ml pancreatic RNase). After treatment of proteinase K and extraction with phenol, DNA was precipitated with ethanol, and then dissolved in 1X TE (10 mM Tris–Cl pH 7.5, 1 mM EDTA). The concentration of DNA was determined by OD_260_ using a spectrophotometer. The quality of DNA was checked by agarose gel electrophoresis. Germline DNA was not analysed in this study.

### Microsatellite instability

Microsatellite analysis using fluorescence-labelled primers and an automated DNA sequencer has been described in detail (Oda et al. 1997). Briefly, five human dinucleotide microsatellites, D2S123, D5S107, D10S197, D11S904 and D13S175, were amplified by polymerase chain reaction (PCR). Forward primers were labelled with the fluorescent compound, 6-FAM (6-carboxyfluorescein) or HEX (6-carboxy-2',4,4',5',7,7'-hexachlorofluorescein). *TaKaRa Taq* (TaKaRa Co. Ltd., Tokyo, Japan) was used as a thermostable polymerase. To compare electrophoretic profiles between two samples, 6-FAM-labelled products and HEX-labelled products were mixed and co-electrophoresed in the ABI310 sequencer (Applied Biosystems, Foster City, CA, USA). Data were processed using the GeneScan software (Applied Biosystems).

### MSH2 and MLH1 immunohistochemistry

Tissue specimens were fixed in buffered 10% formaldehyde and embedded in paraffin. Prior to the assay, the specimens were sectioned at 4 µm and deparaffinised using xylene. Immunohistochemistry was performed using HISTOFINE SAB-PO(M)/(R) Kit (NICHIREI CORPORATION, Tokyo, Japan). Three independent antibodies against MSH2 and MLH1 were used to confirm the results. Expression of PCNA was also used as a positive control for nuclear staining. Antibodies used were as follows: anti-MSH2: Ab-1, Ab-2 (Oncogene Research Products, Cambridge, MA, USA) and MSH-2 (BD Biosciences Pharmingen, Hamburg, Germany); anti-MLH1: Ab-1 (Oncogene Research Products), MLH-1 (BD Biosciences Pharmingen) and C-20 (Santa Cruz Biotechnology Inc., Santa Cruz, CA, USA); anti-PCNA: PC10 (DakoCytomation California Inc., Carpinteria, CA, USA); non-specific IgG: X931 and X936 (DakoCytomation California Inc.).

### Statistical analysis

The cumulative survival curves were plotted using the Kaplan–Meier method and compared using the log rank method. To determine the significance of each clinicopathological variable as a prognostic factor, univariate and multivariate analyses were done using Cox proportional hazards models. All statistical calculations were done using the SAS software version 9.3 (SAS Institute, Cary, NC, USA).

## Results

### Detection of microsatellite instability in adult T-cell leukaemia/lymphoma using High-Resolution Fluorescent Microsatellite Analysis (HRFMA)

**Fig. 1:**
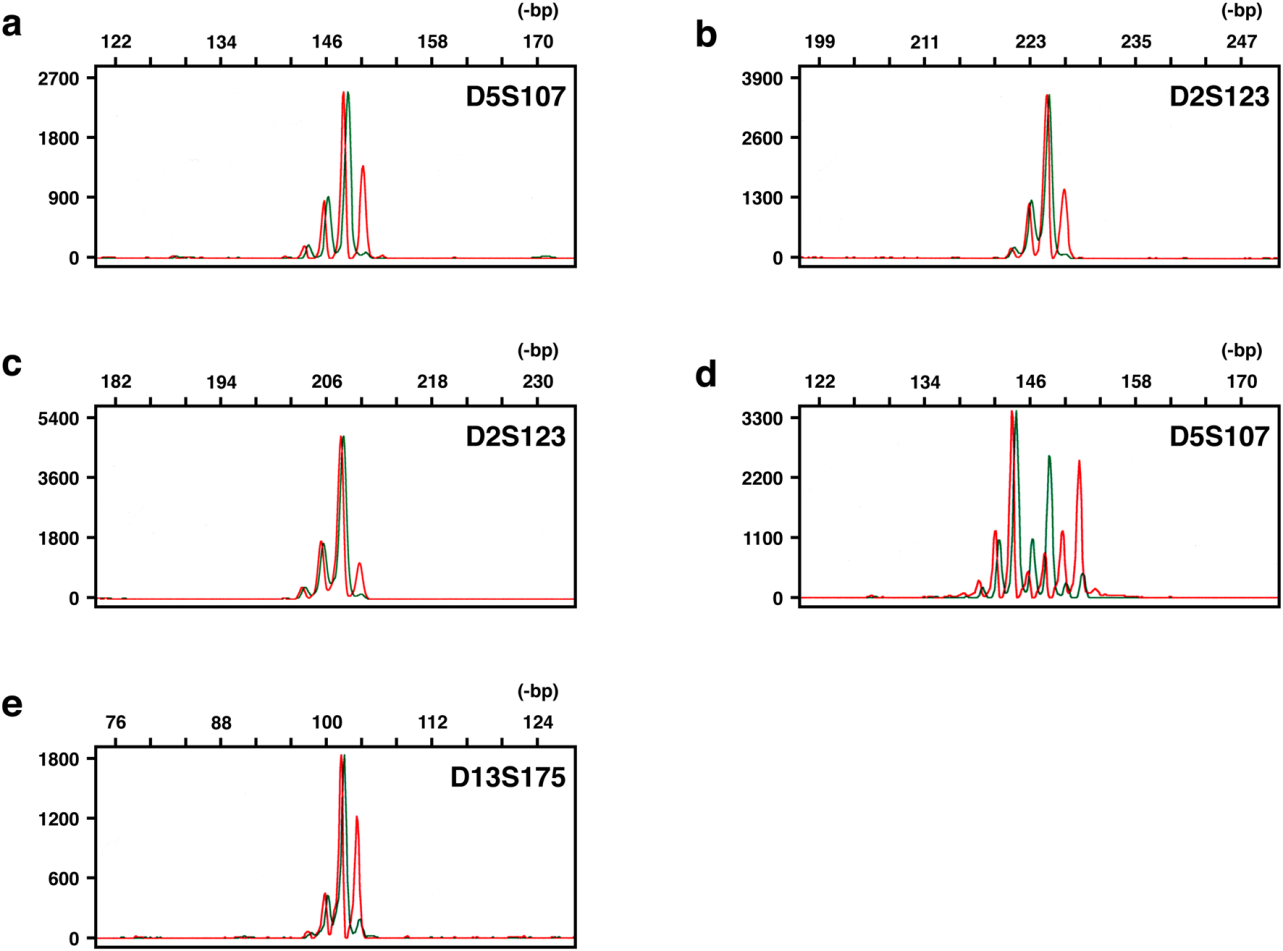
Microsatellite instability observed in adult T-cell leukaemia/lymphoma. The amount of each DNA fragment is quantitatively detected and its size is standardised with an accuracy of one base pair, using co-electrophoresed size markers. Results representative for MSI are shown: red lines, tumour; green lines, normal control. All the microsatellite changes observed were Type A: **a** ATL01; **b** ATL07; **c** ATL09; **d** and **e** ATL16. These patient codes correspond to those in Table 1

We have applied our unique fluorescent technique, HRFMA, for MSI analysis in a panel of 20 ATLL patients (Table 1). Unequivocal alterations in microsatellite sequences were found in four tumours, and are shown in Fig. 1. In three of the four tumours, microsatellite alterations were noted in one of the five markers examined (Table 1), which implies that their phenotypes are classified as MSI-L, according to the NCI criteria (Boland et al. 1998). From another viewpoint, all the observed alterations are categorised as Type A, because the length changes were all within 6-bp (see “Discussion”). More drastic alterations categorised as Type B were not found in this panel. In DNA fragment analyses using a fluorescent system, loss of heterozygosity (LOH) is also detectable, but some patterns of peak clusters are theoretically indistinguishable between Type A MSI and LOH (Fujii et al. 2009). We have designated these peak patters as LOH’. Indeed, LOH’ was observed in several tumours in this panel (Table 1). These tumours were therefore not scored as MSI^+^, which may lead to an underscoring of microsatellite-unstable tumours. Unequivocal LOH was also observed in six tumours of the panel (30%) (Table 1). The above results were highly reproducible in several independent experiments, and thus the overall MSI frequency was determined as 20%.

**Fig. 2:**
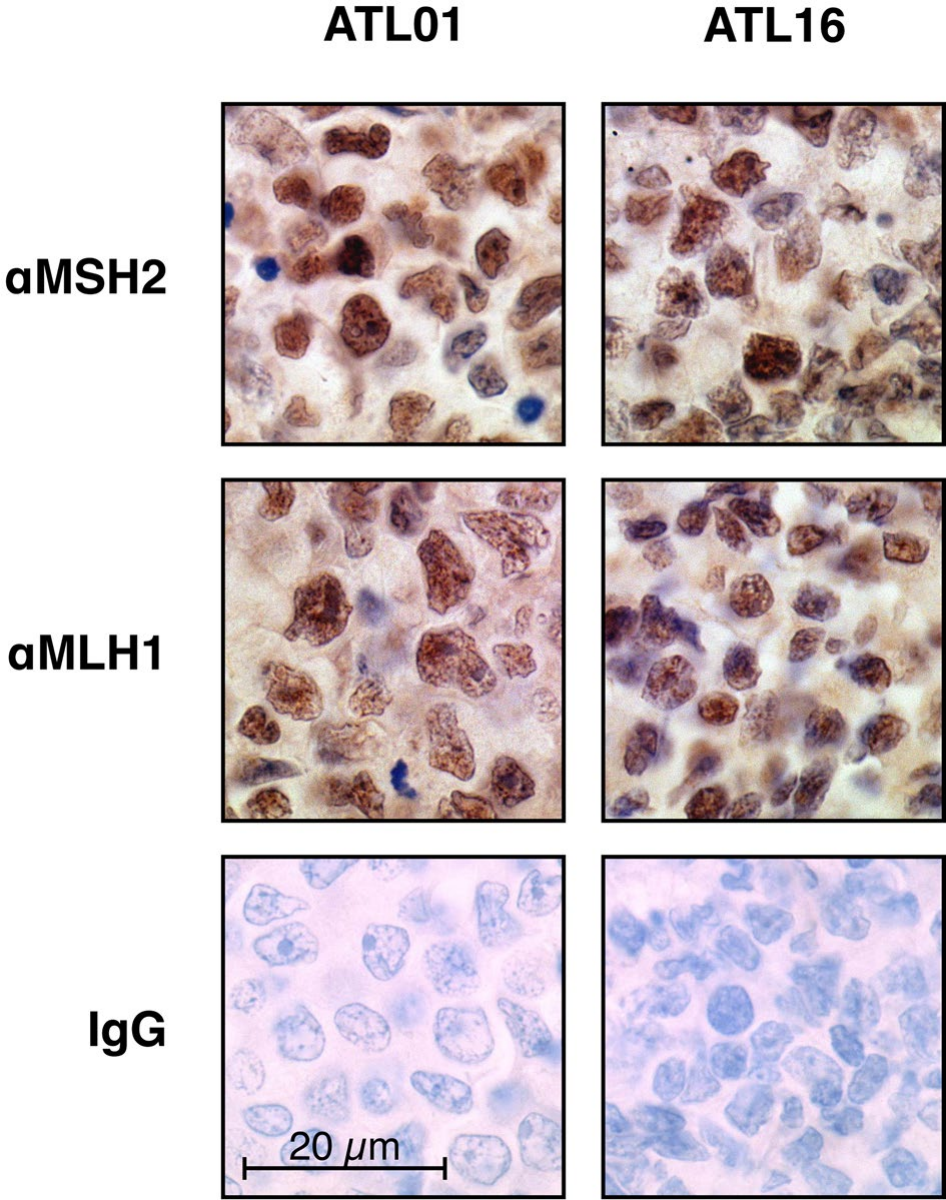
MSH2 and MLH1 immunohistochemistry in MSI^+^ ATLLs. Expressions of MSH2 and MLH1 in the MSI^+^ tumours were examined by immunohistochemistry using three independent antibodies. Representative results are shown (patient ATL01 and ATL16). No abnormal expression of these proteins was observed in all MSI^+^ ATLLs

It is widely known that MSI is often caused by promoter methylation of an essential MMR gene, *MLH1*, particularly in sporadic colorectal carcinomas (Herman et al. 1998). Therefore, we next examined expression of MLH1 in the tumours of the panel, using immunohistochemistry. Loss of MLH1 expression was however observed in none of those including four MSI^+^ tumours (Fig. 2). This observation is consistent with the results previously reported in the literature that *MLH1* silencing by promoter methylations is very infrequent in ATLL tumours (Matsushita et al. 2005). Expression of *MSH2*, another essential MMR gene, was also examined, but no alterations were found (Fig. 2).

### Poor clinical outcomes in adult T-cell leukaemia/lymphoma patients with MSI^+^ tumours

**Fig. 3:**
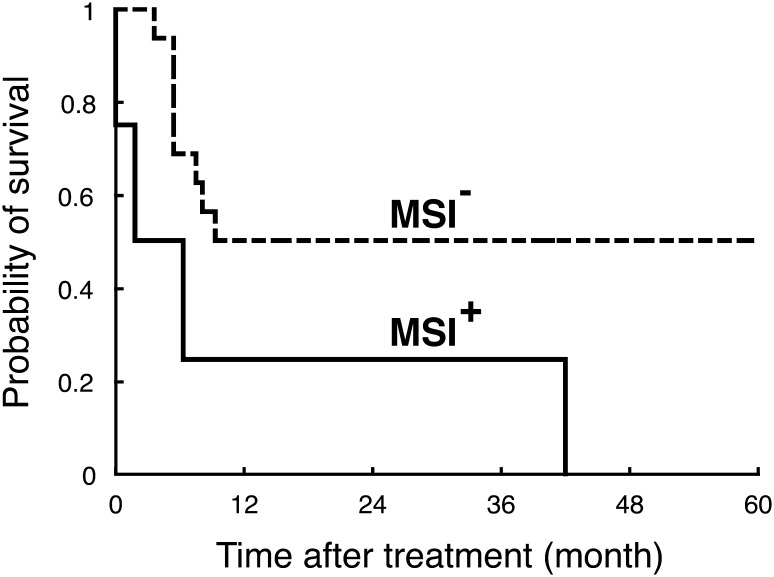
Overall survival of ATLL patients with/without MSI. Cumulative overall survival for each group was obtained using the Kaplan–Meier method

Although HSCT (Jabbour et al. 2011) and target-based therapies (Ishida et al. 2012; Yamamoto et al. 2010) are now regarded as promising, antineoplastic agents still play a major role in the treatment of ATLL patients (Ishitsuka and Tamura 2014; Tsukasaki and Tobinai 2014; Utsunomiya et al. 2015). As mentioned above, MMR deficiency causes cellular resistance against various antineoplastic agents such as cyclophosphamide, doxorubicin (Drummond et al. 1996; Fink et al. 1998) and etoposide (Aebi et al. 1997; Fink et al. 1998). Since these agents are used in the chemotherapy against ATLL, drug resistance and subsequent poor patient prognosis due to defective MMR are of particular interest. Therefore, clinical outcomes were compared between in patients with Type A MSI^+^ tumours and in those without the tumour phenotype. The clinicopathological variables, including age, gender, subtype, Ann Arbor stage, Eastern Cooperative Oncology Group performance status (PS), serum LDH and calcium and number of lesions, were not significantly different between the two groups (data not shown) (see Table 1). Cumulative overall survival for the patients was then determined using the Kaplan–Meier method (Fig. 3). As expected, the patient survival was significantly worse in the MSI^+^ group (*p* = 0.041), and all the four patients succumbed to the disease within four years. Similar results were obtained also in the patient survival before HSCT (*p* = 0.048, data not shown). We next examined whether MSI is an independent prognostic factor. In univariate analyses, *p* values were calculated in each clinicopathological variables, and those for age, PS and MSI were 0.067, 0.058 and 0.054, respectively (Table 2). Finally, multivariate analysis revealed that age (*p* = 0.030) and MSI (*p* = 0.019) are an independent prognostic factor, although, due to the paucity of subjects and events, the 95% confidence interval was broad. Thus, we conclude that Type A MSI is an independent prognostic factor in ATLL patients.

**Table 2:** Univariate and multivariate analyses of prognostic factors in ATLL patients

Variable	Univariate analysis		Multivariate analysis	
	HR (95% CI)	*p* value	HR (95% CI)	*p* value
MSI	3.296 (0.979–11.092)	0.054	5.460 (1.327–22.465)	0.019
Age	1.063 (0.996–1.134)	0.067	1.093 (1.009–1.183)	0.030
Gender (female *vs.* male)	0.326 (0.071–1.494)	0.149		
Subtype (A *vs.* L)	0.460 (0.124–1.704)	0.245		
Stage	1.165 (0.530–2.563)	0.703		
PS (2–4 *vs.* 0–1)	7.266 (0.933–56.560)	0.058		
Hypercalcaemia	1.249 (0.392–3.981)	0.707		

*A* acute type, *CI* confidence interval, *HR* hazard ratio, *L* lymphoma type, *MSI* microsatellite instability, *PS* Eastern Cooperative Oncology Group performance status, *Stage* Ann Arbor stage

## Discussion

To date, MSI in ATLL has been addressed by several groups (Table 3). The first report of microsatellite-unstable ATLLs appeared in the literature in 1998 (Hatta et al. 1998). In this report, Hatta et al. observed the MSI phenomenon in ten out of 22 tumours of their panel (45%), and the observed phenotypes were all MSI-L. In another early report, Hayami et al. (Hayami et al. 1999) found eight microsatellite-unstable tumours in 18 patients (44%), and the frequencies for MSI-H and -L were both 22%. In these two studies, authentic mono- and dinucleotide microsatellite sequences were analysed using autoradiography techniques. On the other hand, the other reports focused genes that carry microsatellite-like repetitive motifs, i.e. mononucleotide runs, in their ORFs, such as *TGFBR2*, *IGF2R*, *BAX*, *E2F4*, *MSH3*, *CASP5*, *TCF4* and *PTEN*. This may be based on the widely accepted viewpoint that destabilisation of the intragenic mononucleotide runs is frequently observed in microsatellite-unstable tumours, particularly in colorectal carcinomas (Schwartz et al. 1999). However, instability of these genes is exclusively associated with the MSI-H phenotype and is very rare in MSI-L or microsatellite-stable (MSS) tumours. This is one reasoning for the two mutually exclusive pathways in colorectal tumourigenesis (Lengauer et al. 1998; Perucho 1996). Indeed, alterations in the repeat-including genes were infrequent in ATLLs. Ohshima et al. (Ohshima et al. 2000) observed no mutations in these genes, whereas Komatsu et al. (Komatsu et al. 2000) reported alterations only in *E2F4* (2/10, 20%) and Takeuchi et al. (Takeuchi et al. 2003) found *TCF4* and *PTEN* mutations in cell lines. These previous observations in the literature may suggest that the MSI phenomenon is indeed observable in ATLLs, and that the MSI-L phenotype may predominate in them. One problem in the first two studies is that the techniques used were gel electrophoresis and autoradiography. In gel electrophoresis, the migration of DNA fragments is error-susceptible and sometimes misread, which will lead to scoring of false positives. Because autoradiography has biased (i.e. non-linear) detection characteristics, the signal of each band is often misestimated and sometimes not detected, which leads to scoring of false negatives. Currently, fluorescent systems using automated DNA sequencers are instead used and prevalent worldwide. We have first applied a fluorescent system for analysing classical intergenic microsatellites in ATLL. Indeed, the assay techniques used for MSI analyses have been in confusion, and several methodological problems are pointed out in them (Maehara et al. 2001). Application of fluorescent systems has remarkably improved the above-mentioned problem in the detection characteristics. We further added dual colour co-electrophoresis to our fluorescent system, in order to completely exclude migration errors. In this system, two differentially labelled PCR products, derived from tumour (red) and the corresponding normal tissues (green), are co-electrophoresed (see Fig. 1). In addition to the problems in the migration and the detection of DNA fragments, the post-synthetic modification of PCR products by thermostable DNA polymerases is also pointed out (Maehara et al. 2001). We therefore employed the enzymatic treatment or the primer sequence modification, in order to simplify electrophoretic profiles of PCR products and consequently to improve the reproducibility of results. Thus, the major methodological problems are overcome in our fluorescent system, HRFMA. Compared with recently tested genome-wide approaches using next-generation sequencers (NGS) (Lu et al. 2013), one disadvantage of PCR-based approaches may be that a very limited number of microsatellites are assayed, and that, therefore, their selection is critical. We have selected five sensitive dinucleotide microsatellites located in five independent chromosomes that are readily destabilised in MMR-defective cells (Oki et al. 1999). We have first applied this unique assay technique for MSI analyses of ATLLs, and the obtained frequency of MSI was 20%.

**Table 3:** Microsatellite instability in ATLL: reports in the literature

Author	Year	No. of cases	Subject		Locus	No. of loci	Detection	MSI		
			Tumour	Control				%MSI	%H	%L
Hatta et al.	1998	22	Unknown	PB	di-	54	R	45	0	45
Hayami et al.	1999	18	PB or LN	Hair root	mono-/di-/tri-/tetra-	6	R	44	22	22
Ohshima et al.	2000	10	Unknown	Placenta	*TGFBR2*, *IGF2R*, *BAX*	3	F	0	0	0
Komatsu et al.	2000	10 (all MSI +)	Unknown	–	*TGFBR2*, *IGF2R*, *BAX*, *E2F4*, *MSH3*	5	Seq	–	–	–
Takeuchi et al.	2003	11	Cell line	–	mono-	1	R	18	18	NE

*di*- dinucleotide microsatellites, *F* fluorescence, *LN* lymph nodes, *mono*- mononucleotide microsatellites, *MSI* microsatellite instability, *NE* not evaluated, *PB* peripheral blood, *R* autoradiography, *Seq* sequencing, *tetra*- tetranucleotide microsatellites, *tri*- trinucleotide microsatellites, *%H* MSI-H (high), *%L* MSI-L (low)

One important finding of this study is that Type A MSI predominates in ATLLs. This may be consistent with the above discussion that the MSI-L phenotype may be predominant in ATLLs, since in colorectal cancer Type A instability tends to be observed in a limited number of markers and, therefore, categorised as MSI-L (Ikeda et al. 2001). Indeed, the majority of the observed microsatellite alterations were noted in one marker (see Table 1). In other words, the MSI-L phenotype was predominant. Nevertheless, the mode of microsatellite alterations appears more important than the frequency of changes, because the relationship between the mode and defective MMR has been approached in our previous study. Type A MSI is defined a relatively small change within 6-bp in dinucleotide microsatellites (i.e. 3 repeat units), and Type B as a more extensive microsatellite change involving ≥ 8-bp (i.e. ≥ 4 repeat units) (Oda et al. 2005). In addition, repeat length alterations in Type B MSI are sometimes discontinuous and, therefore, appear as if a ‘third’ allele is present in addition to the parental alleles. Intriguingly, the microsatellite changes observed in cells of MMR gene-knockout mice and tumours occurring in the animals were invariably Type A, and there was no evidence of the emergence of Type B alterations in the MMR-defective animals (Oda et al. 2005). These observations strongly suggest that defective MMR is necessary and sufficient for Type A instability and that, in other words, Type A MSI is a direct consequence of MMR deficiency. On the other hand, MMR defects may be insufficient for the development of more drastic Type B alterations, and molecular abnormalities in addition to repair deficiency may contribute to, or be responsible for, Type B MSI. Although Type B instability is widely observed in human tumours including the classical examples in hereditary non-polyposis colorectal cancer (HNPCC) (*alias* Lynch syndrome, LS), previously unrecognised molecular abnormalities may underlie tumourigenesis in this category of human neoplasms. Thus, we demonstrated that Type A instability reflects MMR-defective phenotypes in tumour cells. The presence of Type A MSI in ATLLs strongly suggests that MMR deficiency does occur in this malignancy, and that some ATLLs do exhibit the MMR-defective phenotypes.

Another important phenotype of MMR-defective cells is drug resistance. Connection between MMR defects and cellular resistance to anticancer drugs has initially been pointed out by Karran and colleagues (Branch et al. 1993) in 1993. They have shown that cells defective of MMR are highly resistant to mono-functional alkylating agents. Detailed mechanism of drug resistance in repair-deficient cells is still not well understood. It has however been established that a form of methylated guanine bases, i.e. *O* ^6^-methylguanine (O6meG), can pair not only with cytosine but also with thymine, and that the thymine–guanine base mismatch stimulates cellular MMR (Karran 2001). The genome-wide activation of MMR may lead to catastrophic repair reactions on the genome, and, consequently, to cell death. Resistance of MMR-defective cells to 6-thioguanine (6-TG) or 6-mercaptopurine (6-MP) is similarly explained (Swann et al. 1996). Various antineoplastic agents, including 5-FU (Aebi et al. 1997; Carethers et al. 1999; Tokunaga et al. 2000a), cisplatin (Aebi et al. 1996; Anthoney et al. 1996; Drummond et al. 1996), camptothecin, etoposide (Aebi et al. 1997; Fink et al. 1998) and anthracyclines (Drummond et al. 1996; Fink et al. 1998), have been tested since then, and, in some, their effects have been shown to be modulated by cellular MMR activities. In particular, the connection between MMR and 5-FU has attracted attention, since 5-FU is one of the most widely used antineoplastic agents in cancer chemotherapy. It has been demonstrated by several groups that MMR-defective cells are resistant to 5-FU in vitro (Carethers et al. 1999; Tokunaga et al. 2000a), although detailed mechanisms of the resistance are still unknown. This hypothesis, however, has not been verified in the clinical setting. In order to elucidate the connection between defective MMR and tumour resistance to 5-FU, numerous clinical studies have been done, particularly in colorectal cancer patients treated with 5-FU-based post operative adjuvant chemotherapies. However, the data reported in the literature are inconsistent and, therefore, still not conclusive (Guastadisegni et al. 2010). In addition to the complexity of the clinical outcomes of patients, the heterogeneity of the MSI phenomenon in colorectal cancer may, at least partly, contribute to the discrepancy between the in vitro and the in vivo data, since the MSI phenomenon has been uniformly regarded as a hallmark of MMR defects. The absence of Type B MSI in ATLLs may indicate that the relationship between repair defects and the MSI phenomenon is straightforward in this malignancy, and that, in other words, ATLLs exhibiting MSI (i.e. Type A) can be straightforwardly regarded as MMR-defective. Indeed, patient outcomes were significantly worse in MSI^+^ ATLLs, which may be because the tumours had the drug-resistant MMR-defective phenotypes, and chemotherapy was not effective in these patients. Furthermore, we first demonstrated that MSI is an independent prognostic factor in ATLL, although statistical power was limited because of the paucity of subjects and events. These observations strongly support the possibility that Type A MSI is a prognostic and potentially predictive biomarker in ATLL patients, because their clinical outcomes are largely dependent on the results of initial chemotherapy. Thus, in contrast to colorectal cancer, the simplicity of the MSI phenomenon in this malignancy may have led to a more straightforward connection between MSI and drug resistance. Although HSCT and the anti-CCR4 therapy are widely tested, these modalities are used as a second-line treatment, and chemotherapy using antineoplastic agents still plays a major role and is the first line in the treatment of ATLL patients (Ishitsuka and Tamura 2014; Tsukasaki and Tobinai 2014; Utsunomiya et al. 2015). However, there are in fact some tumours that exhibit a remarkable resistance to chemotherapy, as exemplified in the MSI^+^ cases in this study. Biomarkers predicting the tumour sensitivity to antineoplastic agents are of particular importance and use. The Type A MSI phenotype of ATLLs may provide critical information in the treatment strategies. Needless to say, before concluding this, it is necessary to test the hypothesis in a larger cohort, in order to confirm and generalise the results of this study. Propagation of precise assay techniques is also necessary, because Type A alterations are minor and more subtle than the widely acknowledged mode of microsatellites changes (i.e. Type B MSI) and obviously difficult to be detected by less sensitive techniques. These efforts may lead to the establishment of reliable predictive biomarkers and, consequently, to truly personalised approaches for more effective treatment of ATLL patients.

## References

[CR1] Aebi S (1996). Loss of DNA mismatch repair in acquired resistance to cisplatin. Cancer Res.

[CR2] Aebi S, Fink D, Gordon R, Kim HK, Zheng H, Fink JL, Howell SB (1997). Resistance to cytotoxic drugs in DNA mismatch repair-deficient cells. Clin Cancer Res.

[CR3] Anthoney DA, McIlwrath AJ, Gallagher WM, Edlin AR, Brown R (1996). Microsatellite instability, apoptosis, and loss of p53 function in drug-resistant tumor cells. Cancer Res.

[CR4] Araki K (2004). Frequent loss of heterozygosity but rare microsatellite instability in oesophageal cancer in Japanese and Chinese patients. Oncology.

[CR5] Arisawa K (2000). Evaluation of adult T-cell leukemia/lymphoma incidence and its impact on non-Hodgkin lymphoma incidence in southwestern Japan. Int J Cancer.

[CR6] Boland CR (1998). A National Cancer Institute Workshop on Microsatellite Instability for cancer detection and familial predisposition: development of international criteria for the determination of microsatellite instability in colorectal cancer. Cancer Res.

[CR7] Branch P, Aquilina G, Bignami M, Karran P (1993). Defective mismatch binding and a mutator phenotype in cells tolerant to DNA damage. Nature.

[CR8] Carethers JM, Chauhan DP, Fink D, Nebel S, Bresalier RS, Howell SB, Boland CR (1999). Mismatch repair proficiency and in vitro response to 5-fluorouracil. Gastroenterology.

[CR9] Drummond JT, Anthoney A, Brown R, Modrich P (1996). Cisplatin and adriamycin resistance are associated with MutLalpha and mismatch repair deficiency in an ovarian tumor cell line. J Biol Chem.

[CR10] Eto T (2016). Modal variety of microsatellite instability in human endometrial carcinomas. J Cancer Res Clin Oncol.

[CR11] Fink D, Nebel S, Norris PS, Aebi S, Kim HK, Haas M, Howell SB (1998). The effect of different chemotherapeutic agents on the enrichment of DNA mismatch repair-deficient tumour cells. Br J Cancer.

[CR12] Fisher RI (1993). Comparison of a standard regimen (CHOP) with three intensive chemotherapy regimens for advanced non-Hodgkin’s lymphoma. N Engl J Med.

[CR13] Fujii K (2009). Simulation-based analyses reveal stable microsatellite sequences in human pancreatic cancer. Cancer Genet Cytogenet.

[CR14] Goel A (2003). Characterization of sporadic colon cancer by patterns of genomic instability. Cancer Res.

[CR15] Guastadisegni C, Colafranceschi M, Ottini L, Dogliotti E (2010). Microsatellite instability as a marker of prognosis and response to therapy: a meta-analysis of colorectal cancer survival data. Eur J Cancer.

[CR16] Hatta Y, Yamada Y, Tomonaga M, Miyoshi I, Said JW, Koeffler HP (1998). Microsatellite instability in adult T-cell leukaemia. Br J Haematol.

[CR17] Hawkins NJ, Tomlinson I, Meagher A, Ward RL (2001). Microsatellite-stable diploid carcinoma: a biologically distinct and aggressive subset of sporadic colorectal cancer. Br J Cancer.

[CR18] Hayami Y (1999). Microsatellite instability as a potential marker for poor prognosis in adult T cell leukemia/lymphoma. Leuk Lymphoma.

[CR19] Herman JG (1998). Incidence and functional consequences of hMLH1 promoter hypermethylation in colorectal carcinoma. Proc Natl Acad Sci USA.

[CR20] Hishizawa M (2010). Transplantation of allogeneic hematopoietic stem cells for adult T-cell leukemia: a nationwide retrospective study. Blood.

[CR21] Ikeda Y, Oda S, Abe T, Ohno S, Maehara Y, Sugimachi K (2001). Features of microsatellite instability in colorectal cancer: comparison between colon and rectum. Oncology.

[CR22] Ishida T (2012). Defucosylated anti-CCR4 monoclonal antibody (KW-0761) for relapsed adult T-cell leukemia-lymphoma: a multicenter phase II study. J Clin Oncol.

[CR23] Ishitsuka K, Tamura K (2014). Human T-cell leukaemia virus type I and adult T-cell leukaemia-lymphoma. Lancet Oncol.

[CR24] Jabbour M (2011). Hematopoietic SCT for adult T-cell leukemia/lymphoma: a review. Bone Marrow Transplant.

[CR25] Jass JR, Walsh MD, Barker M, Simms LA, Young J, Leggett BA (2002). Distinction between familial and sporadic forms of colorectal cancer showing DNA microsatellite instability. Eur J Cancer.

[CR26] Jiricny J (2006). The multifaceted mismatch-repair system. Nat Rev Mol Cell Biol.

[CR27] Kagami Y (1996). Feasibility of salvage chemotherapy for refractory or relapsed non-Hodgkin’s lymphoma with two topoisomerase II inhibitors, MST-16 and VP-16. MST-16 Study Group. Int J Hematol.

[CR28] Kanda J (2012). Impact of graft-versus-host disease on outcomes after allogeneic hematopoietic cell transplantation for adult T-cell leukemia: a retrospective cohort study. Blood.

[CR29] Karran P (2001). Mechanisms of tolerance to DNA damaging therapeutic drugs. Carcinogenesis.

[CR30] Komatsu N (2000). Mutations of the E2F4 gene in hematological malignancies having microsatellite instability. Blood.

[CR31] Lengauer C, Kinzler KW, Vogelstein B (1998). Genetic instabilities in human cancers. Nature.

[CR32] Lu Y, Soong TD, Elemento O (2013). A novel approach for characterizing microsatellite instability in cancer cells. PLoS ONE.

[CR33] Maehara Y, Oda S, Sugimachi K (2001). The instability within: problems in current analyses of microsatellite instability. Mutat Res.

[CR34] Matsuoka M, Jeang KT (2007). Human T-cell leukaemia virus type 1 (HTLV-1) infectivity and cellular transformation. Nat Rev Cancer.

[CR35] Matsushita M (2005). Methylation of the MLH1 gene in hematological malignancies. Oncol Rep.

[CR36] Miyashita K (2008). Frequent microsatellite instability in non-Hodgkin lymphomas irresponsive to chemotherapy. Leuk Res.

[CR37] Modrich P (2006). Mechanisms in eukaryotic mismatch repair. J Biol Chem.

[CR38] Oda S, Oki E, Maehara Y, Sugimachi K (1997). Precise assessment of microsatellite instability using high resolution fluorescent microsatellite analysis. Nucleic Acids Res.

[CR39] Oda S (2005). Two modes of microsatellite instability in human cancer: differential connection of defective DNA mismatch repair to dinucleotide repeat instability. Nucleic Acids Res.

[CR40] Ohshima K (2000). Mutation analysis of mitotic checkpoint genes (hBUB1 and hBUBR1) and microsatellite instability in adult T-cell leukemia/lymphoma. Cancer Lett.

[CR41] Okamura J (2005). Allogeneic stem-cell transplantation with reduced conditioning intensity as a novel immunotherapy and antiviral therapy for adult T-cell leukemia/lymphoma. Blood.

[CR42] Oki E, Oda S, Maehara Y, Sugimachi K (1999). Mutated gene-specific phenotypes of dinucleotide repeat instability in human colorectal carcinoma cell lines deficient in DNA mismatch repair. Oncogene.

[CR43] Perucho M (1996). Microsatellite instability: the mutator that mutates the other mutator. Nat Med.

[CR44] Sakurai M, Zhao Y, Oki E, Kakeji Y, Oda S, Maehara Y (2007). High-resolution fluorescent analysis of microsatellite instability in gastric cancer. Eur J Gastroenterol Hepatol.

[CR45] Schwartz S, Yamamoto H, Navarro M, Maestro M, Reventos J, Perucho M (1999). Frameshift mutations at mononucleotide repeats in caspase-5 and other target genes in endometrial and gastrointestinal cancer of the microsatellite mutator phenotype. Cancer Res.

[CR46] Shimoyama M (1991). Diagnostic criteria and classification of clinical subtypes of adult T-cell leukaemia-lymphoma. A report from the Lymphoma Study Group (1984–87). Br J Haematol.

[CR47] Swann PF, Waters TR, Moulton DC, Xu YZ, Zheng Q, Edwards M, Mace R (1996). Role of postreplicative DNA mismatch repair in the cytotoxic action of thioguanine. Science.

[CR48] Takeuchi S, Takeuchi N, Fermin AC, Taguchi H, Koeffler HP (2003). Frameshift mutations in caspase-5 and other target genes in leukemia and lymphoma cell lines having microsatellite instability. Leuk Res.

[CR49] Tokunaga E, Oda S, Fukushima M, Maehara Y, Sugimachi K (2000). Differential growth inhibition by 5-fluorouracil in human colorectal carcinoma cell lines. Eur J Cancer.

[CR50] Tokunaga E (2000). Frequency of microsatellite instability in breast cancer determined by high-resolution fluorescent microsatellite analysis. Oncology.

[CR51] Tsukasaki K, Tobinai K (2014). Human T-cell lymphotropic virus type I-associated adult T-cell leukemia-lymphoma: new directions in clinical research. Clin Cancer Res.

[CR52] Tsukasaki K (2007). VCAP-AMP-VECP compared with biweekly CHOP for adult T-cell leukemia-lymphoma: Japan Clinical Oncology Group Study JCOG9801. J Clin Oncol.

[CR53] Tsukasaki K (2009). Definition, prognostic factors, treatment, and response criteria of adult T-cell leukemia-lymphoma: a proposal from an international consensus meeting. J Clin Oncol.

[CR54] Uchiyama T (1997). Human T cell leukemia virus type I (HTLV-I) and human diseases. Annu Rev Immunol.

[CR55] Uchiyama T, Yodoi J, Sagawa K, Takatsuki K, Uchino H (1977). Adult T-cell leukemia: clinical and hematologic features of 16 cases. Blood.

[CR56] Utsunomiya A (2001). Improved outcome of adult T cell leukemia/lymphoma with allogeneic hematopoietic stem cell transplantation. Bone Marrow Transplant.

[CR57] Utsunomiya A, Choi I, Chihara D, Seto M (2015). Recent advances in the treatment of adult T-cell leukemia-lymphomas. Cancer Sci.

[CR58] Verdonck K, Gonzalez E, Van Dooren S, Vandamme AM, Vanham G, Gotuzzo E (2007). Human T-lymphotropic virus 1: recent knowledge about an ancient infection. Lancet Infect Dis.

[CR59] Wilson WH (1993). EPOCH chemotherapy: toxicity and efficacy in relapsed and refractory non-Hodgkin’s lymphoma. J Clin Oncol.

[CR60] Yamada Y (2001). A new G-CSF-supported combination chemotherapy, LSG15, for adult T-cell leukaemia-lymphoma: Japan Clinical Oncology Group Study 9303. Br J Haematol.

[CR61] Yamamoto K (2010). Phase I study of KW-0761, a defucosylated humanized anti-CCR4 antibody, in relapsed patients with adult T-cell leukemia-lymphoma and peripheral T-cell lymphoma. J Clin Oncol.

[CR62] Yonekura K, Utsunomiya A, Takatsuka Y, Takeuchi S, Tashiro Y, Kanzaki T, Kanekura T (2008). Graft-versus-adult T-cell leukemia/lymphoma effect following allogeneic hematopoietic stem cell transplantation. Bone Marrow Transplant.

[CR63] Young J (2001). Features of colorectal cancers with high-level microsatellite instability occurring in familial and sporadic settings: parallel pathways of tumorigenesis. Am J Pathol.

